# Endothelial progenitor cell levels in juvenile idiopathic arthritis patients; effects of anti-inflammatory therapies

**DOI:** 10.1186/s12969-015-0001-4

**Published:** 2015-02-19

**Authors:** Malgorzata Rusak, Urszula Radzikowska, Barbara Glowinska-Olszewska, Elzbieta Dobrenko, Janina Piotrowska-Jastrzebska, Milena Dabrowska, Anna Bodzenta-Lukaszyk, Artur Bossowski, Marcin Moniuszko

**Affiliations:** Department of Hematological Diagnostics, Medical University of Bialystok, 15-274 Bialystok, Poland; Department of Regenerative Medicine and Immune Regulation, Medical University of Bialystok, 15-269 Bialystok, Poland; Department of Pediatrics, Endocrinology, Diabetology with Cardiology Division, Medical University of Bialystok, 15-274 Białystok, Poland; Department of Pediatrics and Developmental Disorders, Medical University of Bialystok, 15-274 Białystok, Poland; Department of Allergology and Internal Medicine, Medical University of Bialystok, 15-276 Bialystok, Poland

**Keywords:** Endothelial progenitor cells, Juvenile idiopathic arthritis, Cardiovascular risk factors, Anti-inflammatory therapies

## Abstract

**Background:**

Juvenile idiopathic arthritis (JIA), similarly to other arthritides, can be associated with damage of endothelial layer of which structure and function is dependent on reparative properties of endothelial progenitor cells (EPC). To date, it remained unknown whether EPC numbers are altered in young JIA patients and whether on-going anti-inflammatory therapies could exert positive effects on these progenitor cells.

**Methods:**

We performed a quantitative analysis of EPC numbers in 25 patients diagnosed with JIA according to International League of Associations for Rheumatism (ILAR) criteria [age 11.50 (7.50-15.00) years] in a broad context of inflammatory and cardiovascular parameters as well as different types of anti-inflammatory treatments. 11 healthy children [age 13.00 (11.00-14.00) years] were recruited as a control group.

**Results:**

We demonstrated that EPC numbers were similar in JIA patients and control subjects (0.02% vs. 0.05%, respectively, p = 0.37). EPC levels in JIA patients were negatively correlated with index of insulin resistance (rho = -0.458, p = 0.021), endogenous insulin (rho = -0.472, p = 0.017), triglyceride (rho = -0.438, p = 0.029) and TNF-alpha levels (rho = -0.446, p = 0.026). Notably, glucocorticoid (GC) therapy, was associated with detection of decreased EPC levels in JIA patients (p = 0.023). In contrast, methothrexate (MTX) and etanercept therapy in JIA patients did not affect EPC levels (p = 0.92 and p = 0.08, respectively).

**Conclusions:**

We found that EPC numbers are maintained at normal levels in JIA patients and are not enhanced by disease-specific anti-inflammatory treatments.

## Background

Rheumatoid arthritis (RA) is chronic inflammatory disease that is associated with significantly increased risk of development of cardiovascular diseases (CVD) [1]. Patients with RA suffer from 2-5-fold higher morbidity and mortality related to CVD which in turn leads to the shortened (by 5-10 years) life expectancy [2]. Development of CVD in RA patients is caused by progressive endothelial dysfunction and damage [3]. Preservation of well-functioning endothelium, its appropriate repair and regeneration is warranted by continuous supplementation of endothelial progenitor cells (EPC) [2]. EPC originate from bone marrow, circulate in peripheral blood and keep responding to signals initiated by endothelial injury that stimulate maturation of EPC to new endothelial cells [4]. Thus, EPC remain a key regulator of vascular maintenance and repair [2,3,5,6]. Indeed, decreased numbers and/or impaired function of EPCs in adult RA patients were associated with significant endothelial dysfunction leading to accelerated atherosclerosis and development of CVD [6-8]. However, studies on quantification of circulating EPC numbers brought controversial results as several groups reported either enhanced or unaltered EPC levels in adult RA patients [9-11].

In contrast to RA, much less is known about the role of EPC in the pathogenesis of juvenile idiopathic arthritis (JIA) associated with cardiovascular system injury. JIA is the most frequently occurring rheumatic disease in children [12,13]. Despite early onset of disease, JIA was characterized by development of vascular damage early in childhood long before establishment of diagnosis of clinically apparent cardiovascular diseases [12]. Similarly to RA, we recently have demonstrated an increased cardiovascular risk in JIA patients [14]. Accordingly, Coulson *et al.* proposed that long-term risk of development of CVD in JIA patients could be even higher than in other adult-onset inflammatory arthritides [12]. Therefore, the studies on therapies aiming at decreasing CVD risk in JIA patients are still warranted. Decrease in CVD risk could be achieved by improvement of endothelial layer structure and function which, in turn, is dependent on reparative properties of EPC [4,15]. Recently, we found that elevated frequencies of EPC in type 1 diabetes children correlated inversely with parameters of endothelial function [16]. Physiologically, children have significantly higher level of endothelial progenitor cells than older individuals [17]. To date, it remained unknown whether numbers of EPC could be altered in young JIA patients.

In the current study we performed analysis of EPC in samples derived from these patients as we wished to investigate putative changes in EPC numbers in the course of JIA. We aimed to identify potential inflammatory and cardiovascular parameters that could be related to these changes. Finally, we set out to analyse whether administration of anti-inflammatory treatment could result in alterations of EPC levels similar to those seen in adult RA patients.

## Methods

### Study participants

We recruited 25 children, 12 (48%) girls and 13 (52%) boys, aged 11.50 (7.50-15.00) years, diagnosed with juvenile idiopathic arthritis (according to International League of Associations for Rheumatism criteria) for at least one year; they were followed at the tertiary academic center, Medical University of Bialystok, Poland. Oligoarticular and polyarticular types of JIA were reported in 14 (56%) and 11 (44%) patients respectively. No child had been recognized with systemic type of the disease. Children were divided into clinically active (n = 13 – 54%) and inactive (n = 11 - 46%) based on current practice recommendations [18]. The recruitment for the study group, all clinical examinations and qualification to the groups were performed by an experienced pediatric rheumatologist (ED). The control group included healthy normal-weight boys (n = 4) and girls (n = 7) aged 13.00 (11.00-14.00) years with blood pressure below 90^th^ percentile according to reference, negative family history of CVD and absence of systemic inflammatory disease based on physical and laboratory examination. All children underwent physical examination, height and weight were taken in a standard way using Harpenden stadiometer and digital scale (Seca, Germany). Body mass index (BMI) was calculated with a standard formula. Overweight was determined when the BMI (kg/m^2^) exceeded the 85^th^ centile whereas obesity as BMI exceeding the 95^th^ centile according to national growth references [19]. Because the BMI is not normally distributed in childhood, we used the least mean square method, which normalizes the BMI skewed distribution and expresses BMI as an standard deviation score (SDS-BMI). Systolic (SBP) and diastolic (DBP) blood pressures were measured twice at the right arm after a 10-minute rest using calibrated sphygmomanometer with appropriate cuff size, and were averaged. We obtained approval of the Ethical Committee in the Medical University of Bialystok. Both parents/legal guardians and children gave their written informed consent.

### Laboratory investigations

Blood sample of 10 mL was taken from the left cubital vein, after an overnight (8-12 hr) fast. To assess inflammatory markers serum samples were collected, frozen and stored at the temperature of -80°C until analyses were performed. The concentrations of adiponectin, fractalkine, VE-kadherin, sICAM-1, sVCAM-1, sE-selectin, MMP-2, MMP-9, TIMP-1, osteoprotegerin and interleukins: IL-6, IL-18, TNF-alpha were determined with the use of commercially available ELISA kits (Parameter Human Immunoassays, R&D Systems, Inc., Minneapolis, USA) with the use of ELx 800 Automated Microplate Reader, Bio-Tek Instruments, Vermont, USA. hsCRP was determined with use of immunoturbidymetric method (Tina-quant hsCRP (Latex) HS, Roche; Hitachi 912, La Roche, Japan). Concentrations of lipid, glucose and insulin were determined by standard enzymatic methods (Hitachi 912, La Roche, Japan). LDL concentration was assessed by the Friedewald equation. The homeostasis model was used to assess insulin resistance (HOMA IR) derived from the following formula: insulin resistance (HOMA IR) = (fasting insulin (mU/ml) x fasting glucose (mmol/l))/22.5.

### Flow cytometry analysis

For quantification of EPC, fresh EDTA-anticoagulated whole-blood samples were collected. 110 μl of peripheral blood was immediately stained (30 minutes in room temperature) with 20 μl of anti-human CD34 FITC (BD Pharmingen) and 5 μl of anti-human CD309 (VEGFR-2) PE (BD Pharmingen) monoclonal antibodies. Afterwards samples were lysed with BD FACS Lysing Solution (BD Bioscences), washed twice with PBS and fixed with use of CellFix (BD Biosciences) according to manufacturer’s procedure. Cells were analyzed by FACS Calibur flow cytometer and CellQuest Software (both from BD Immunocytometry Systems). Positive events gates was set via FMO (fluorescence-minus-one) controls. Based on positive surface expression of CD34 EPC were characterized as CD34+ CD309+ (CD34+ VEGFR-2+) cells localized in mononuclear cells (MNCs) gates and shown as percentages of total MNCs.

### Ultrasonographic imagining

Examinations of the carotid and brachial arteries were performed with Hewlett Packard Sonos 4500 apparatus, using a 7.5 MHz linear transducer. The procedure was conducted between 8.00-10.00 AM after 8-12 hours fasting. Measurements of intima-media thickness (IMT) in the common carotid arteries (right and left) were performed as previously described, with own modification [20]. Measurements included end-diastolic (minimum diameter) IMT of the far walls, at the distance of more than 1 cm from the bifurcation. Analyses included the mean value of 6 measurements.

Ultrasound examination of the right brachial arteries was performed in longitudinal sections 2-10 cm above the elbow, according to guidelines [21]. The principle is to induce vasodilatation in the proximal (brachial) artery by post-ischemic (forearm) enhanced flow. All lumen diameter measurements were scanned at end diastole by use of the R-wave of the electrocardiogram. First scans were taken at rest, and second scans during reactive hyperemia. Increased flow was induced by deflating a pneumatic tourniquet placed on the right forearm, inflated to the pressure about 50 mmHg above the patient’s resting systolic blood pressure for 4.5 min. The post-ischemic scan was performed 45-120 seconds after cuff deflation. FMD was derived from the percentage change of the brachial artery diameter after ischemia of the forearm from baseline.

### Statistical analysis

Statistical analysis was performed with use of GraphPad Prism 6 (GraphPad Software). D’Agostino & Pearson omnibus normality test was used to determine normal distribution of data. Normally distributed samples were analyzed with unpaired t-test and are presented as mean ± standard deviation (SD). Mann-Whitney test was used to determine differences in groups do not fitting parameterized distribution, data are presented as median with interquartile range (IQR). To test statistically significant correlations between variables of interest, Spearman’s correlation was used. In all tests, level of statistical significance was set on p < 0.05.

## Results

### Patients

Demographic and clinical characteristics of JIA patients and healthy controls is presented in Table 1. Both groups were similar with regard to gender, age, body mass, height, BMI and SDS BMI. For further analysis study group was divided according to the clinical criteria (activity, type of disease and treatment).

**Table 1:** **General characteristics of study group**

	**JIA patients**	**Control group**	**p**
Number of patients	25	11	
Gender			
Boys (n, %)	13 (52%)	4 (36%)	0.48^+^
Girls (n, %)	12 (48%)	7 (64%)	
Age (years)	11.50 (7.50-15.00)	13.00 (11.00-14.00)	0.46^^^
Body mass (kg)	44.54 ± 18.43	47.23 ± 13.17	0.67^*^
Height (m)	1.48 ± 0.20	1.59 ± 0.12	0.11^*^
BMI (kg/m2)	19.40 (16.65-21.10)	18.60 (15.60-20.60)	0.50^^^
SDS-BMI	0.30 (-0.10-0.85)	.0.15 (-0.60-0.48)	0.33^^^
Insulin (mU/ml)	7.70 (4.00-10.95)	6.60 (3.60-8.70)	0.24^^^
Total-cholesterol (mg/dl)	162.00 ± 26.23	160.20 ± 26.98	0.85^*^
LDL-cholesterol (mg/dl)	80.84 ± 16.90	86.64 ± 24.65	0.42^*^
HDL-cholesterol (mg/dl)	57.40 ± 9.87	56.00 ±11.13	0.71^*^
Triglycerides (mg/dl)	65.00 (49.50-86.50)	64.00 (47.00-100.00)	0.99^^^
SBP (mmHg)	112.3 (98.65-125.20)	112.70 (105.70-121.70)	0.93^^^
DBP (mmHg)	59.98 ± 9.19	68.55 ± 8.75	0.01^*^
FMT (%)	7.96 ± 3.47	10.37 ± 2.63	0.048^*^
IMT (mm)	0.48 ± 0.09	0.45 ± 0.05	0.27^*^
Obesity (n, %)	5 (20%)		
Insulin resistance (n, %)	6 (24%)		
Dyslipidemia (n, %)	7 (28%)		
Age of onset (years)	7.00 (3.50-12.25)		
Disease duration (years)	3.76 ± 2.58		
Disease activity (n, %)			
Clinically active	13 (54%)		
Clinically inactive	11 (46%)		
Type of disease (n, %)			
Oligoarticular	14 (56%)		
Polyarticular	11 (44%)		
Treatment (n, %)			
Glucocorticoids (GC)	18 (72%)		
Etanercept	6 (24%)		
Metothrexate (MTX)	19 (76%)		
Treatment regimens (n,%)			
MTX	4 (16%)		
GC/MTX	9 (36%)		
GC/Etanercept/MTX	5 (20%)		
GC/Sulfasalasine	4 (16%)		

^+^Fisher's exact test. ^*^unpaired t-test. ^^^Mann-Whitney test.

Results are presented as means ± SD or medians with IQR when appropriate and numbers (n) and percentages (%) where required.

### Frequencies of CD34+ and CD34+ CD309+ EPCs in juvenile arthritis patients

**Figure 1:**
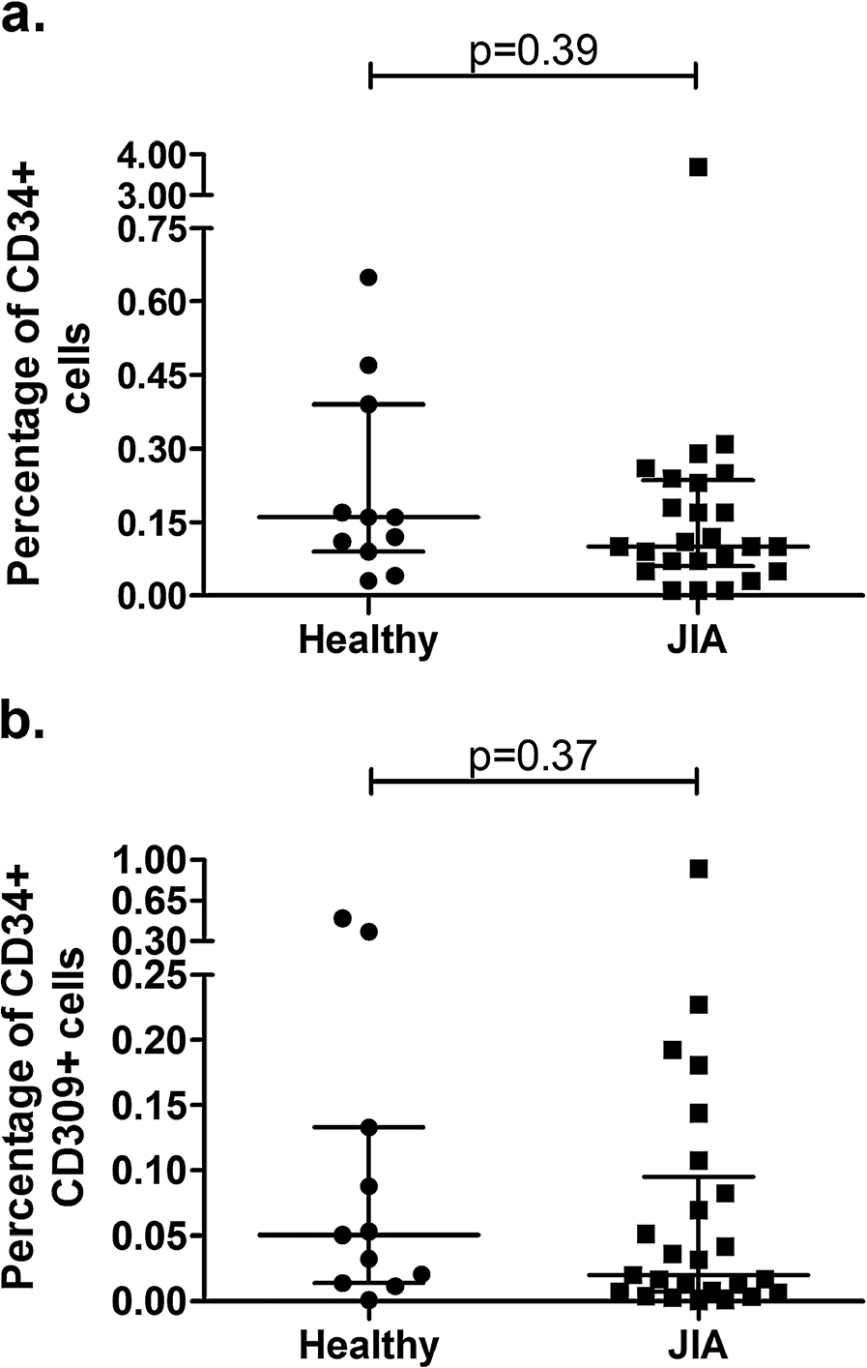
**Frequencies of EPC in JIA patients and healthy controls.** Frequencies of **a)** CD34+ and **b)** CD34+ CD309+ EPC in healthy controls and patients with JIA. Median values are presented as black, horizontal lines while whiskers represent interquartile range (IQR).

Frequencies of CD34+ cells were comparable in JIA patients and control subjects (0.1% [0.06%-0.24%] vs. 0.16% [0.09%-0.39%], respectively, p = 0.39, Figure 1a). Analogously, frequencies of CD34+ VEGFR-2 (CD309) + cells were similar in JIA patients and healthy controls (0.02% [0.007%-0.095%] vs. 0.05% [0.014%-0.13%], respectively, p = 0.37, Figure 1b). Notably, levels of CD34 + CD309+ cells were not related to either activity or type of disease.

### Relationships among CD34+ CD309+ cells in juvenile arthritis patients with clinical and laboratory risk factors for cardiovascular diseases

Next, we wished to evaluate correlations among frequencies of CD34+ CD309+ cells and varying risk factors for CVD development. The majority of CVD risk factors were not significantly associated with levels of CD34 + CD309+ cells (Table 2). Interestingly, however, we demonstrated significant negative correlation between frequencies of CD34 + CD309+ cells and endogenous insulin levels (rho = -0.472, p = 0.017) and HOMA IR (rho = -0.458, p = 0.021). Similar correlation was found for CD34 + CD309+ cells and levels of triglycerides (rho = **-**0.438, p = 0.029) and TNF-α (rho = -0.446, p = 0.026).

**Table 2:** **Correlations of EPC frequencies in patients with JIA and cardiovascular diseases development risk factors**

	**Percentage of CD34 + CD309+ cells**		
**Variable**	**n**	**rho**	**p**
BMI	25	−0.381	0.061
SDS-BMI	25	−0.223	0.283
SBP	25	−0.231	0.266
DBP	25	−0.257	0.216
FMD	25	0.329	0.108
IMT	25	−0.260	0.210
Glucose	25	0.005	0.983
**Insulin**	**25**	**−0.472**	**0.017**
**HOMA IR**	**25**	**−0.458**	**0.021**
**Triglicerides**	**25**	**−0.438**	**0.029**
Total cholesterol	25	−0.267	0.196
LDL-cholesterol	25	0.301	0.144
HDL-cholesterol	25	0.003	0.990
hsCRP	25	−0.210	0.313
Fractalkine	25	−0.031	0.884
Adiponectin	25	0.145	0.488
VE-kadherin	25	−0.045	0.829
sICAM-1	25	−0.148	0.481
sVCAM-1	25	−0.157	0.454
sE-selectin	25	0.009	0.965
MMP-2	25	0.140	0.505
MMP-9	25	0.135	0.519
TIMP-1	25	0.126	0.548
OSP	25	0.159	0.447
**TNF-α**	**25**	**−0.446**	**0.026**
IL-18	25	−0.364	0.074
IL-6	25	−0.184	0.380

rho: Spearman's correlation coefficient.

Statistically significant correlations are presented in bold. n: number of patients.

### Effects of glucocorticoid, etanercept and methotrexate treatment on EPC levels in JIA patients

**Figure 2:**
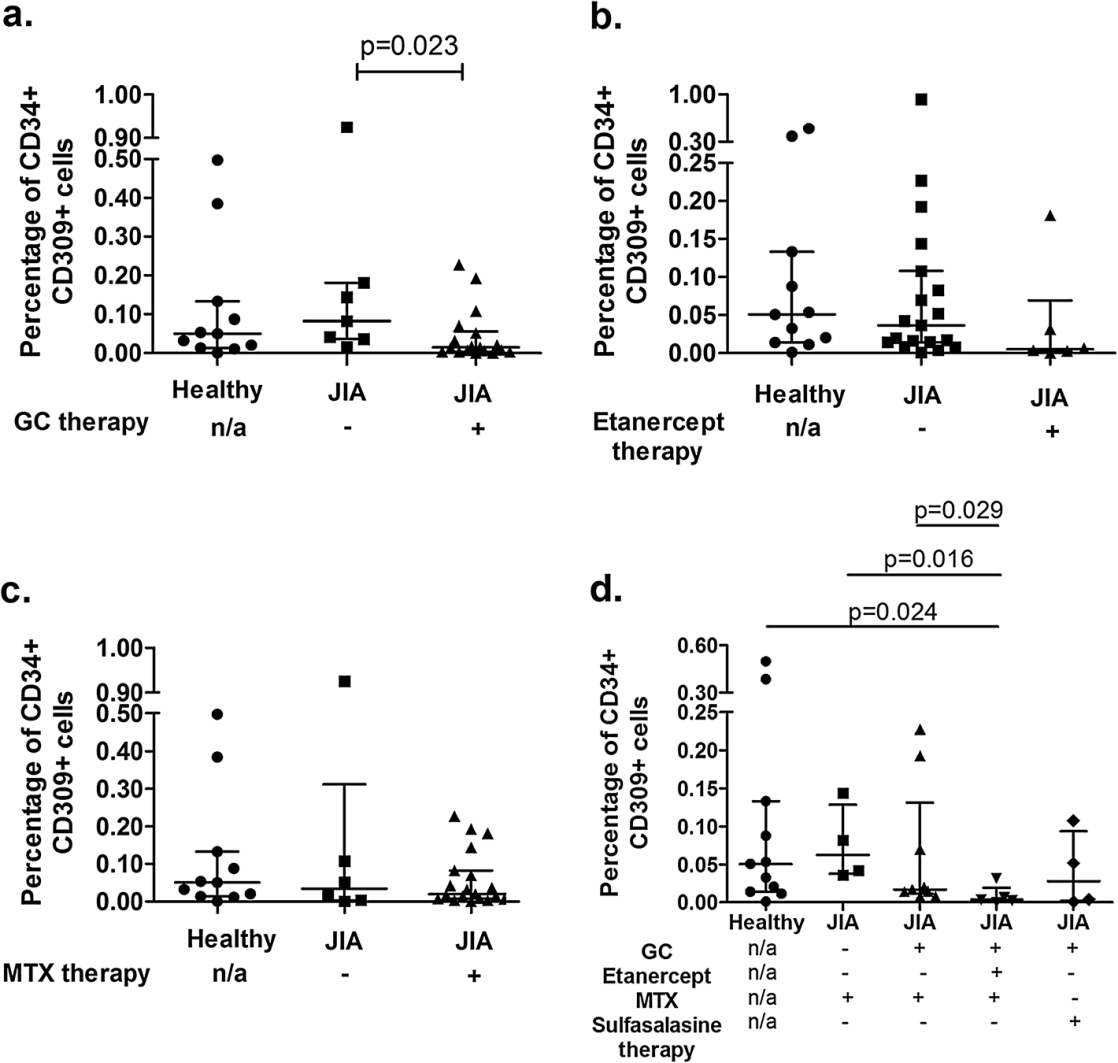
**Frequencies of EPC in JIA patients undergoing anti-inflammatory therapies.** Frequencies of CD34 + CD309+ EPC in JIA patients undergoing **a)** glucocorticoid (GC), **b)** etanercept, **c)** methotrexate (MTX) therapy. **d)** Summary of the effects of different treatment regimens on frequencies of CD34 + CD309+ cells. Median values are presented as black, horizontal lines, while whiskers represent IQR. Statistically significant differences are shown; n/a: not applicable (healthy controls).

Furthermore, we demonstrated that patients with JIA that were treated with GC demonstrated lower levels of CD34 + CD309+ cells as compared to patients not treated with GC (0.015% [0.004%-0.06%] vs. 0.08% [0.037%-0.18%]; p = 0.023, respectively; Figure 2a). In some contrast, levels of CD34 + CD309+ cells in JIA patients treated or not with etancercept were similar (0.005% [0.002%-0.07%] vs. 0.04% [0.014%-0.11%]; p = 0.08, respectively; Figure 1b).

Next, we demonstrated that metothrexate therapy in JIA patients did not affect levels of CD34 + CD309+ cells (0.02% [0.007%-0.08%] vs. 0.03% [0.003%-0.31%]; p = 0.92, for JIA patients with and without MTX, respectively) (Figure 2c).

Having found that different anti-inflammatory drugs can exert differential effects on EPC levels, we wished to analyse whether these effects can be attributed to different treatment schemes (Figure 2d). We demonstrated that lowest CD34 + CD309+ cell levels were found in these JIA patients who received combination of GC, etanercept and metothrexate (0.0035% [0.0015%-0.019%] vs. 0.05% [0.014%-0.13%]; p = 0.024 for JIA and healthy subjects, respectively). Interestingly, JIA patients treated with GC, etanercept and metothrexate presented with significantly lower EPC levels than patients treated with metothrexate alone (0.0035% [0.0015%-0.019%] vs. 0.062% [0.038%-0.13%], respectively; p = 0.016) or patients treated with combination of GC and metothexate (0.0035% [0.0015%-0.019%] vs. 0.017% [0.011%-0.13%], respectively; p = 0.029) (Figure 2d).

## Discussion

Here we performed a cross-sectional analysis of EPC numbers in JIA patients in a broad context of inflammatory and cardiovascular parameters and on-going anti-inflammatory treatment. In clear contrast to studies performed in adult RA patients, we demonstrated that EPC numbers in JIA pediatric patients are at similar levels as compared to healthy children. Our study supplements the data derived from adult patients with arthritis. Previous studies revealed that adult RA patients at mean age of 34 years presented with insignificantly decreased EPC levels [8]. In some contrast, elder RA patients at mean age of 54 years were found to have significantly decreased EPC levels that, in addition, were correlated to disease activity [7]. In our study, however, we did not find relationships between EPC numbers and disease activity. Given our and other published data, one could hypothesize that EPC in arthritis patients are preserved only at early stages of disease and then they are becoming gradually diminished in the course of following years.

One of hypotheses explaining decreased EPC levels observed in long-lasting RA patients relates this alteration to enhanced neovascularization taking place in inflamed joints. Indeed, neovascularization remains a major culprit accounting for synovial hyperplasia observed in the course of both, RA and JIA [13,22]. Notably, substantial numbers of EPC have been detected in synovial tissue of RA patients [23]. Therefore, reduced levels of EPC observed in peripheral blood of adult RA patients could be related to enhanced angiogenesis in synovium which in turn stimulates increased migration of EPC to inflamed joints. This however has to be accompanied by an efficient homeostatic regeneration and recirculation of newly developing EPC. Given our data showing unaltered EPC levels in pediatric JIA patients and data demonstrating decreased EPC levels in adult RA patients, one cannot exclude that in the course of age the capacity to renew decreasing peripheral pool of EPC might be diminished.

Chronic systemic inflammation being characteristic feature of JIA affects efficient regeneration of endothelial cells of remodelled arteries, which in turn leads to development of atherosclerosis and increased risk of CVD. In the current study we correlated EPC numbers with parameters associated with increased CVD risk. Quite surprisingly, we found only few significant correlations between EPC and CVD risk factors (e.g. endogenous insulin, HOMA IR and TNF-alpha). This finding indicates that augmentation of pro-inflammatory mediators in JIA patients does not seem to be as strongly related to changes in EPC levels as in adult RA patients. Moreover, we did not find significant relationships among EPC numbers and parameters of development of atherosclerosis, namely FMD and IMT. These data are in contrast to our recent observations of type 1 diabetes children with early signs of vascular damage whose EPC numbers were significantly inversely correlated to FMD [16]. It also has been shown, that EPC correlate inversely with cIMT in young patients with long-standing type 1 diabetes [24]. This suggests that interplay between EPC and vascular damage represents a more complex phenomenon that could be affected by specific disease-related factors. Interestingly, however, we found that EPC levels were negatively related to levels of endogenous insulin and HOMA IR. Both abovementioned parameters were found to constitute relevant risk factors for development of CVD [25,26]. Notably, this correlation was found despite the fact that endogenous insulin levels in JIA patients were comparable to those seen in healthy controls. However, 24% of JIA patients developed insulin resistance, which stays in contrary with control group and may indicate that our data are also in concert with reports demonstrating that insulin-resistance was associated with reduced EPC numbers in animal model of human normoglycemia [27]. In some parallel, insulin-like growth factor 1 (IGF-1) was recently demonstrated to decrease survival of certain subsets of adult stem cells [28-31]. Our demonstration of negative relationship among EPC and insulin concentrations suggests that insulin could exert negative effects on EPC generation and/or distribution. However, in some contrast, Humpert *et al*. demonstrated that formation of EPC colony-forming units derived from human PBMCs was enhanced by IGF-1 receptor-dependent signalling [32]. Moreover, Zhao *et al*. reported that moderate but not higher concentrations of insulin and glucose promoted EPC proliferation [33]. Altogether, it may suggest that dependent on concentration and microenvironment, insulin could exert varying effects on levels and function of circulating EPC.

To date, changes in distribution of EPC in JIA patients were not evaluated in the context of ongoing therapies. Current management of JIA is, similarly to RA, based on introduction of varying regimens based on administration of anti-inflammatory drugs including methotrexate, glucocorticoid and anti-TNF-alpha monoclonal antibodies. Again, vast majority of current data on potential interactions between applied treatment of arthritides and EPC derive from adult RA patients. Surprisingly, to date, the interactions between EPC and methotrexate were not studied in sufficient detail. Grisar *et al.* reported, that active RA patients treated with MTX showed similar EPC frequencies to patients did not receive MTX therapy [7]. Additionally, it has been shown, that methotrexate may induce apoptosis of EPC derived from healthy volunteers [8]. However, those *in vitro* studies require confirmation, considering impaired function of EPC in rheumatoid patients, which may affect influence of methotrexate on EPC numbers. On the other hand, former studies in RA patients revealed that both GC and etanercept bear capacity to enhance decreased EPC levels [34,35]. With regard to GC, the positive effects on EPC numbers were found only for intermediate, but not low doses of the drug [34]. In some contrast, in the current study, we did not observe positive effects any of anti-inflammatory treatments applied in JIA patients on EPC numbers. Moreover, we demonstrated that in our group of patients, GC therapy was associated with decrease in EPC levels. Intriguingly, this effect was most pronounced in these patients who were treated with combination of GC, metothrexate and etanercept in some contrast to patients treated with GC and metothrexate. This finding provides another, yet unexplored example of complex interactions between traditional anti-inflammatory drugs and biologic response modifiers. One could hypothesize that lack of enhancement of EPC numbers by etanercept or GC could be related to the fact that JIA patients managed to preserve sustainable EPC numbers. On the other hand, however, one cannot exclude that normal levels of EPC observed in studied JIA patients could have resulted from administered anti-inflammatory therapies, and without GC therapy associated with systemic inflammation EPC level in JIA patients could have been lower. Our present understanding of dynamic interactions between pool of circulating EPC and those cells that are attracted to inflamed joints is very limited. Therefore, demonstration of decreased EPC levels in peripheral blood can be also explained by their migration to inflamed joints. Similarly, lack of enhancement of circulating EPC in pediatric patients following anti-inflammatory treatment could be a result of accerelated distribution of these cells to inflamed synovial tissues. Further studies exploring mutual relationships between circulating and synovial EPC are still warranted. Nonetheless, comparative analysis of our and other studies indicates that similar treatment regimens introduced to patients at different age can exert differential effects on EPC numbers. In addition, given our data, future would shed more light on complex effects of drugs used in JIA on regenerative potential of JIA patients.

## Conclusions

Altogether, we demonstrated here that EPC levels are maintained at normal levels in pediatric JIA patients and they are not significantly enhanced by on-going anti-inflammatory therapies.

## Abbreviations

BMI: Body mass index
CVD: Cardiovascular disease
DBP: Diastolic blood pressure
EPC: Endothelial progenitor cell
FMD: Flow-mediated dilatation
GC: Glucococorticoid
HOMA IR: Index of insulin resistance
hsCRP: High sensitive C-reactive protein
ILAR: International League of Associations for Rheumatism
IMT: Intima-media thickness
JIA: Juvenile idiopathic arthritis
MMP-2: Matrix metaloproteinase-2
MMP-9: Matrix metaloproteinase-9
MTX: Methotrexate
OSP: Osteoprotegerin
PBMC: Peripheral blood mononuclear cells
RA: Rheumatoid arthritis
SDS BMI: Standard deviation score body mass index
sE-selectin: Soluable E-selectin
sICAM-1: Soluable ICAM-1
SPB: Systolic blood pressure
sVCAM-1: Soluable VCAM-1
TIMP-1: Tissue inhibitor of metalloproteinases-1
